# Nutritional health attitudes and behaviors and their associations with the risk of overweight/obesity among child care providers in Michigan Migrant and Seasonal Head Start centers

**DOI:** 10.1186/s12889-016-3328-y

**Published:** 2016-07-27

**Authors:** Won O. Song, SuJin Song, Violeta Nieves, Andie Gonzalez, Elahé T. Crockett

**Affiliations:** 1 Department of Food Science and Human Nutrition, Michigan State University, East Lansing, MI 48824 USA; 2 Department of Medicine-College of Human Medicine, Michigan State University, East Lansing, MI 48824 USA

**Keywords:** Nutritional health behavior, Overweight, Obesity, Migrant and Seasonal Head Start, Child care provider, Childhood obesity

## Abstract

**Background:**

Children enrolled in Migrant and Seasonal Head Start (MSHS) programs are at high risks of health problems. Although non-family child care providers play important roles on children’s health status as role models, educators, program deliverers, and information mediators, little is known about their nutritional health attitudes and behaviors, and weight status. Therefore, we investigated nutritional health attitudes and behaviors and their associations with overweight/obesity among child care providers in Michigan MSHS centers.

**Methods:**

A total of 307 child care providers aged ≥ 18 years working in 17 Michigan MSHS centers were included in this cross-sectional study conducted in 2013. An online survey questionnaire was used to collect data on nutritional health attitudes and behaviors of child care providers. Weight status was categorized into normal weight (18.5 ≤ BMI < 25 kg/m^2^), overweight (25 ≤ BMI < 30 kg/m^2^), and obese (BMI ≥ 30 kg/m^2^) based on child care providers’ self-reported height and weight. Factor analysis was performed to investigate patterns of nutritional health attitudes and behaviors. Multivariate logistic regression was conducted to estimate the odds ratios (ORs) and 95 % confidence intervals (CIs) of overweight/obesity across tertiles of pattern scores taking the lowest tertile group as the reference group after adjustment for potential confounding variables.

**Results:**

Three patterns of nutritional health attitudes and behaviors were identified: pattern 1) “weight loss practices with weight dissatisfaction”, pattern 2) “healthy eating behaviors”, and pattern 3) “better knowledge of nutrition and health”. The pattern 1 scores were positively associated with overweight/obesity (Tertile 2 vs. Tertile 1: OR = 5.81, 95 % CI = 2.81–12.05; Tertile 3 vs. Tertile 1: OR = 14.89, 95 % CI = 6.18–35.92). Within the pattern 2, the OR for overweight/obesity in individuals with the highest scores was 0.37 (95 % CI = 0.19–0.75) compared with those with the lowest scores. However, the pattern 3 was not associated with the risk of overweight/obesity.

**Conclusions:**

Our findings support that nutrition education or health interventions targeting MSHS child care providers are urgently necessary. These efforts might be an efficient and effective approach for improving the nutritional health status of young children enrolled in MSHS programs.

## Background

A Migrant and Seasonal Head Start (MSHS) provides comprehensive early childhood education services for children ages zero to five years from Migrant and Seasonal Farm Worker (MSFW) families to promote school readiness and to help them grow physically, mentally, emotionally, and socially [1]. It was established in 1969 to respond to the needs of MSFWs [1], who are individuals employed in agricultural works on a seasonal basis with or without moving from their permanent residence [2]. The MSHS program differs from a regular Head Start program in that the participants being MSFWs’ children, longer operation hours and remote rural locations, and programs staffed dominantly with Spanish speaking members [1, 3]. The MSHS programs also offer health and nutrition related services, including nutritious meals and nutrition education to improve the overall health status of children [1]. In 2013, the MSHS program served 31,907 MSFWs’ children nationwide [1].

Since MSFWs’ children spend long hours with child care providers in MSHS centers, child care providers in MSHS centers working with children play an important role in the nutritional health status of MSFWs’ children. Non-family child care providers are known to have the significant impact on the prevention of childhood obesity through role modeling of nutritional health behaviors and body image, teaching and practicing healthful dietary habits, implementing nutrition and health programs, and mediating information related to nutrition and health for families, parents, and children [4, 5].

However, only a few studies have examined non-family child care provider’s nutritional health behaviors and their associations with child’s health outcomes [6-8]. Regular Head Start teachers in Texas showed unhealthy dietary habits, such as low consumption of fruits and vegetables, high consumption of fried foods and soda and thus had a high prevalence of overweight/obesity [8]. Child care providers in licensed child care programs in rural Southern Illinois had low nutrition knowledge and inappropriate child feeding behaviors at mealtime [7]. In addition, feeding behaviors of child care providers working in Head Start in Texas were associated with children’s food consumption [6]. Little is known about the nutritional health attitudes and behaviors among child care providers in MSHS programs.

Child care providers in MSHS centers are of particular importance due to their unique needs and great impact on the nutritional health status in MSFWs’ children, who are vulnerable to overweight/obesity [9–11]. In addition, identifying patterns of nutritional health attitudes and behaviors can capture the complex nature of nutritional health attitudes and behaviors based on their inter-correlations and provide a comprehensive approach to explore their relationships with health outcomes. Understanding nutritional health attitudes and behaviors and their associations with weight status among MSHS child care providers is the first step to develop intervention strategies to improve their health status and ability to deliver MSHS programs to young children. Ultimately, these efforts might lead to improve the nutritional health status among MSFWs’ children through a positive role modeling and successful implementation of MSHS programs by child care providers. Therefore, the aim of this study was to investigate patterns of nutritional health attitudes and behaviors and examine their associations with overweight/obesity among child care providers in Michigan MSHS centers.

## Methods

### Study design and participants

Michigan Telamon Corporation provides MSHS services to young children aged zero to five years from MSFW families in 18 centers throughout the state. To be eligible for MSHS services, primary source of family income must come from qualifying agricultural activities and qualify based on income guidelines. Michigan MSHS centers offers partial or full day services with season varies from 10–26 weeks but primarily runs from June through October [12].

In the summer of 2013, Michigan Telamon Corporation collaborated with a research team in the Department of Food Science and Human Nutrition at Michigan State University to conduct a nutritional needs assessment for the overall goal of improving its MSHS programs and addressing the nutritional health risks that impact Michigan MSFW’s children. This needs assessment was necessary in order to learn about the current nutritional health status of Michigan MSFW’s children and how this is influenced by external factors, including sociodemographic characteristics, weight status, weight related perception and behaviors, nutritional health attitudes and behaviors, nutrition knowledge, and food availability and security of their parents and child care providers in MSHS centers. Our previous work examined the parental risk factors of childhood overweight/obesity in this population [11].

To answer the questions arisen from this study, data collected from child care providers who worked in 17 Michigan MSHS centers were used. The child care providers were defined as all employees worked in MSHS centers, including teachers and other staff who were aged 18 years or older. Study participants were recruited through an email in English and Spanish. The email indicated the instruction about the needs assessment and was disseminated to directors of all registered Michigan MSHS centers which had about 407 available child care providers. A total of 311 child care providers participated in this study (response rate: 76.4 %). Among them, two participants who had incomplete data on food availability and food security status and two participants who were underweight were excluded. Underweight is associated with other distorted eating behaviors related to anorexia and weight perceptions [13, 14], so we did not include them in the reference group of normal weight to avoid any potential problem with data interpretation occurring from inclusion of underweight participants. In addition, a very small number of participants were in the underweight category. Therefore, 307 child care providers were included in the final data analysis. We provided financial supports for a catered lunch to the participating centers as compensation. Our research team provided this compensation to the participating centers after the data collection was completed to avoid any potential bias in the answers from child care providers given in this study. However, each participant did not receive any incentives of participating in this study. The formal approval to conduct this needs assessment was obtained by the Institutional Review Board of the Michigan Telamon Corporation. Informed written consent was obtained from each child care provider for collecting data by the research team.

### Data collection

To collect data in this study, an online survey questionnaire was developed by nutritional professionals of the research team using an advanced and user-friendly online survey software tool (SurveyGizmo, Boulder, CO, USA). The completed computerized survey data were submitted via-online directly into a secured database, with limited access to those coordinating this project. The survey questionnaire link was sent via-email to each center and then distributed by each MSHS director to their child care providers. Prior to the dissemination of survey questionnaire link, we held a meeting with center directors to enhance their familiarity to this study, including the purposes, procedures, and the survey questionnaires. Directors guided their child care providers how to complete the survey questionnaire at each center. This self-administered survey given to child care providers in Michigan MSHS centers was completed within two weeks after its dissemination.

The survey questionnaire was divided into 1) sociodemographic characteristics, 2) weight status, 3) perception of weight, 4) nutrition knowledge, 5) food availability, 6) food security status, and 7) nutritional health attitudes and behaviors. Questions on sociodemographic characteristics included gender, age, race/ethnicity, marital status, and education level. Height and weight of child care providers were measured using calibrated portable scales that were located in MSHS centers by child care providers and then self-administered to the questionnaire. Body mass index (BMI) was calculated as weight (in kg) divided by height squared (in m^2^). Weight status was categorized into normal weight (18.5 ≤ BMI < 25 kg/m^2^), overweight (25 ≤ BMI < 30 kg/m^2^), and obese (BMI ≥ 30 kg/m^2^) based on the definition of overweight/obesity from the Centers for Disease Control and Prevention [15]. Perception of their weight was answered as underweight, normal weight, overweight, or obese. Nutrition knowledge was evaluated using nine questions which were adopted from a part of the Head Start on Healthy Living Teacher Health Behavior Survey questionnaire validated and used in the previous study for Head Start teachers in Texas [8]. Nine questions were 1) Do drinks, like Fruitopia or Sunny Delight, count as a fruit serving?, 2) Do only fresh fruits and vegetables count towards the recommended daily servings of fruit and vegetables?, 3) Is it okay for children to eat without worrying about fat because they need lots of extra calories to grow?, 4) Are soft drinks low in fat?, 5) Are dairy products a good source of calcium?, 6) Should vitamin and mineral supplements be taken in addition to a healthy diet?, 7) How many servings of fruits and vegetables should you eat per day?, 8) What percent of your daily calories should come from fat?, and 9) What has the most calories?. A score of one was assigned to questions answered correctly, and a zero to a wrong answer or do not know response. The sum of the nutrition knowledge scores was the total nutrition knowledge score.

Food availability was assessed based on six questions related to the cost, quality, and accessibility of food, which were adopted from a part of the questionnaire used in the previous study examining the family food environments [16]. Food security status of adults was evaluated based on the 2012 US household food security survey module and was divided into four categories according to raw score: 1) high food security (score = 0), 2) marginal food security (score 1–2), 3) low food security (score 3–5), and 4) very low food security (score 6–10) [17]. Fifteen questions regarding nutritional health attitudes and behaviors were adopted from a part of the Teens Eating for Energy and Nutrition at School teaching staff survey [18, 19] and answered as five-scale from strongly disagree to strongly agree.

### Statistical analyses

All statistical analyses were conducted using SAS version 9.3 (SAS Institute Inc., Cary, NC, USA). To identify specific patterns of nutritional health attitudes and behaviors among child care providers, Principal Component Analysis with varimax rotation (PROC FACTOR and VARIMAX options in SAS) was performed based on 15 questions related to nutritional health attitudes and behaviors. As an input for factor analysis, the five-scale answers from strongly disagree to strongly agree for these questions were converted into continuous values from 1 to 5 to derive patterns. The output of factor analysis included all the eigenvalues, the factor loading matrix for eigenvalues greater than one, and computed factor scores. Factor scores for each pattern were calculated as the weighted sum scores by multiplying the score of each question into its factor loading and then summing all of them and the score for each identified pattern was given to each individual. To determine which patterns to retain, the eigenvalue, the factor loading matrix, and interpretability were considered [20]. The derived patterns were interpreted and named according to nutritional health attitudes and behaviors based on the questions with higher factor loadings (≥ |0.40|) in each identified pattern. Because of non-normal distribution of pattern scores, individuals were categorized into three groups by tertiles of scores for each pattern.

Sociodemographic characteristics, weight status, perception of weight, nutrition knowledge level, food availability, and food security status across the tertiles of pattern scores were presented as means and standard deviation (SD) for continuous variables and as percentages (%) for categorical variables. These variables across the tertiles of pattern scores were compared using the general linear model for continuous variables and the chi-square test for categorical variables. Multivariate logistic regression was performed to estimate odds ratios (ORs), 95 % confidence intervals (CIs), and *p*-values for the prevalence of overweight/obesity across the tertiles of pattern scores, taking the lowest tertile group as the reference group after adjustment for gender (male or female), age (<30y, 30–49y, or ≥ 50y), race/ethnicity (White/Caucasian, Black/African American, Hispanic/Latino, or other), marital status (single, married, cohabitating, or separated/divorced/widowed), and education level (≤ high school or ≥ Associate’s degree/certificate or college) as potential confounding variables. All statistical tests were two-sided, and a *p*-value < 0.05 represented statistical significance.

## Results

### Characteristics of Michigan MSHS child care providers

Characteristics of Michigan MSHS child care providers are presented in Table 1. Michigan MSHS child care providers included in this study had 37.6 mean age (SD = 13.5). About 92.2 % of them were women and majority was White/Caucasian (44.3 %) or Hispanic/Latino (52.5 %). Child care providers who had associate’s degree/certificate or college degree were 45.6 %. The specific position of child care providers included directors (5.5 %), teachers and assistant teachers (33.6 %), specialists and related workers in education services, family services, food services, health services, and special services (25.4 %), center aide (18.2 %), secretary (8.1 %), bus driver (6.8 %), custodial (1.3 %), and others (1.0 %). The prevalence of overweight/obesity was 73.6 %, but only 9.8 % perceived their weight status as obese. The prevalence of discordance between weight status and perception of weight was about 55 %. The mean score of nutrition knowledge level among child care providers in MSHS centers was 4.1 (SD = 1.4) out of 9. Six questions out of nine were answered correctly by < 50 %. Question 5 (Are dairy products a good source of calcium?) had the highest percentage of child care providers answering correctly (96.4 %) whereas question 8 (What percent of your daily calories should come from fat?) had the lowest percentage answering correctly (8.4 %). The food availability related to cost, quality, and access to grocery store was relatively high but about 28 % reported low or very low food security status in this population.

**Table 1:** Characteristics of child care providers in Michigan Migrant and Seasonal Head Start centers (*n* = 307)

Characteristics	% or Mean(SD)
Gender	
Male	7.8
Female	92.2
Age	
<30y	34.5
30–49y	43.0
≥50y	22.5
Race/Ethnicity	
White/Caucasian	44.3
Black/African American	1.6
Hispanic/Latino	52.5
Other	1.6
Marital status	
Single	30.9
Married	51.1
Cohabitating	6.8
Separated/Divorced/Widowed	11.1
Education level	
≤ High school	54.4
≥ Associate’s degree/certificate or college	45.6
Weight status	
Normal weight	26.4
Overweight	24.1
Obese	49.5
Perception of weight	
Underweight	0.7
Normal weight	30.9
Overweight	58.6
Obese	9.8
Accordance between weight status and perception of weight	
Accordance	45.3
Discordance	54.7
Nutrition knowledge level (% of child care providers who answered correctly)	
Do drinks, like Fruitopia or Sunny Delight, count as a fruit serving?	86.1
Do only fresh fruits and vegetables count towards the recommended daily servings of fruit and vegetables?	36.3
Is it okay for children to eat without worrying about fat because they need lots of extra calories to grow?	83.2
Are soft drinks low in fat?	13.6
Are dairy products a good source of calcium?	96.4
Should vitamin and mineral supplements be taken in addition to a healthy diet?	17.2
How many servings of fruits and vegetables should you eat per day?	29.5
What percent of your daily calories should come from fat?	8.4
What has the most calories?	38.5
Mean(SD) number of corrected answers	4.1 (1.4)
Food availability	
I do not buy many fruits and vegetables because they cost too much.	
Agree	24.4
Neutral	16.3
Disagree	59.3
I do not buy many fruits and vegetables because my family does not like them.	
Agree	3.3
Neutral	6.5
Disagree	90.2
The fresh produce in my area is usually high quality.	
Agree	64.2
Neutral	27.7
Disagree	8.1
It is easy to buy food in my area.	
Agree	78.2
Neutral	14.7
Disagree	7.2
In minutes, how long does it take you to get to the grocery store?	
10 or less	48.5
20	24.4
30	16.3
45 or more	10.8
How many times do you visit the grocery store in a month?	
0–1	3.3
2–4	49.8
5 or more	46.9
Food security status	
High	52.4
Marginal	19.9
Low	16.0
Very low	11.7

### Three patterns of nutritional health attitudes and behaviors

Table 2 shows the factor loading matrix for three patterns of nutritional health attitudes and behaviors identified by factor analysis among Michigan MSHS child care providers. Three patterns accounted for about 57 % of the total variance in the dataset. The first pattern was negatively associated with body weight satisfaction but positively associated with behaviors related to weight loss attempt and was named the “weight loss practices with weight dissatisfaction”. In other words, subjects who had a high score of pattern 1 were more likely to be less satisfied with their body weight and try to lose body weight compared to those who had a low score of the same pattern. The second pattern was characterized by high factor loadings for satisfaction of health and weight status and healthy dietary behaviors, so it was named the “healthy eating behaviors”. The first and second patterns accounted for 21.6 % and 21.4 % of the total variance, respectively. The third pattern was positively associated with the awareness that nutrition is importance for health outcomes for themselves as well as MSHS children and accounted for 13.8 % of the total variance. This pattern was named the “better knowledge of nutrition and health”.

**Table 2:** Factor loading matrix of nutritional health attitudes and behaviors patterns^a^

Nutritional health attitudes and behaviors		Pattern 1 “Weight loss practices with weight dissatisfaction”	Pattern 2 “Healthy eating behaviors”	Pattern 3 “Better knowledge of nutrition and health”
1.	I am in good health.		0.51	
2.	I am satisfied with my weight.	−0.78		
3.	Compared to other adults who are my height, I feel my weight is just right.	−0.71	0.45	
4.	I have tried to lose or gain weight in the past 12 months.	0.78		
5.	I am trying to lose weight now.	0.86		
6.	I am on a special kind of diet, either to lose weight or for health-related concerns.	0.60		
7.	My eating habits are healthy.		0.81	
8.	I usually limit the amount of high-fat foods I consume.		0.78	
9.	I usually limit the amount of high-sugar items I consume.		0.72	
10.	I usually eat fruits and vegetables daily.		0.57	
11.	I am satisfied with the amount of physical activity I get.		0.50	
12.	What I eat affects my chances of developing disease.			0.72
13.	People who are overweight have a higher risk of health problems.			0.76
14.	Skipping meals affects my ability to do well in the day.			0.69
15.	I can influence the eating behaviors of migrant head start children.			0.61
Variance explained by each factor		21.6	21.4	13.8

^a^The patterns were identified by factor analysis with 15 questions related nutritional health attitudes and behaviors. Factor loadings < | 0.40 | are not shown for simplicity

### Associations of sociodemographic and weight-related characteristics with three patterns

Individuals in the highest tertile of “weight loss practices with weight dissatisfaction” pattern scores were more likely to be older, be obese, and perceive their weight status incorrectly than those in the lowest tertile. The “healthy eating behaviors” pattern scores were not significantly associated with any sociodemographic characteristics. Individuals in the highest tertile of “healthy eating behaviors” pattern scores were more likely to be normal weight and perceive their weight status correctly than those in the lowest tertile. The percentages of White/Caucasian and individuals with high education level (≥ associate’s degree/certificate or college) were significantly higher in the highest tertile of “better knowledge of nutrition and health” pattern scores than those in the lowest tertile. The “better knowledge of nutrition and health” pattern scores were not associated with weight status and perception of weight (Table 3).

**Table 3:** Sociodemographic and weight-related characteristics across the tertiles of scores of nutritional health attitudes and behaviors patterns

	Pattern 1				Pattern 2				Pattern 3			
	“Weight loss practices with weight dissatisfaction”				“Healthy eating behaviors”				“Better knowledge of nutrition and health”			
	Tertile1 (*n* = 102)	Tertile2 (*n* = 103)	Tertile3 (*n* = 102)	*p*-value*	Tertile1 (*n* = 102)	Tertile2 (*n* = 103)	Tertile3 (*n* = 102)	*p*-value*	Tertile1 (*n* = 102)	Tertile2 (*n* = 103)	Tertile3 (*n* = 102)	*p*-value*
Gender (%)												
Male	13.7	2.9	6.9	0.068	7.8	6.8	8.8	0.795	9.8	6.8	6.9	0.435
Female	86.3	97.1	93.1		92.2	93.2	91.2		90.2	93.2	93.1	
Age (%)												
<30y	49.0	29.1	25.5	0.003	39.2	34.0	30.4	0.061	43.1	25.2	35.3	0.260
30–49y	32.4	48.5	48.0		40.2	50.5	38.2		37.3	50.5	41.2	
≥50y	18.6	22.3	26.5		20.6	15.5	31.4		19.6	24.3	23.5	
Race/Ethnicity (%)												
White/Caucasian	44.1	40.8	48.0	0.406	41.2	42.7	49.0	0.239	33.3	46.6	52.9	0.010
Black/African American	1.0	2.9	1.0		2.0	1.0	2.0		1.0	3.9	0.0	
Hispanic/Latino	51.0	55.3	51.0		55.9	52.4	49.0		64.7	48.5	44.1	
Other	3.9	1.0	0.0		1.0	3.9	0.0		1.0	1.0	2.9	
Marital status (%)												
Single	39.2	33.0	20.6	0.066	35.3	28.2	29.4	0.230	39.2	23.3	30.4	0.290
Married	43.1	54.4	55.9		46.1	54.4	52.9		44.1	55.3	53.9	
Cohabitating	9.8	3.9	6.9		5.9	6.8	7.8		7.8	6.8	5.9	
Separated/Divorced/Widowed	7.8	8.7	16.7		12.8	10.7	9.8		8.8	14.6	9.8	
Education level (%)												
≤ High school	52.9	58.3	52.0	0.888	57.8	55.3	50.0	0.262	67.7	52.4	43.1	0.001
≥ Associate’s degree/certificate or college	47.1	41.8	48.0		42.2	44.7	50.0		32.4	47.6	56.9	
Weight status (%)												
Normal weight	52.9	17.5	8.8	<0.001	18.6	24.3	36.3	<0.001	21.6	23.3	34.3	0.279
Overweight	27.5	26.2	18.6		19.6	23.3	29.4		29.4	26.2	16.7	
Obese	19.6	56.3	72.6		61.8	52.4	34.3		49.0	50.5	49.0	
Perception of weight (%)												
Underweight	0.0	1.9	0.0	<0.001	2.0	0.0	0.0	0.002	2.0	0.0	0.0	0.309
Normal weight	69.6	15.5	7.8		18.6	33.0	41.2		33.3	24.3	35.3	
Overweight	29.4	77.7	68.6		64.7	58.3	52.9		60.8	61.2	53.9	
Obese	1.0	4.9	23.5		14.7	8.7	5.9		3.9	14.6	10.8	
Accordance between weight status and perception of weight (%)												
Accordance	63.7	29.1	43.1	0.003	41.2	39.8	54.9	0.049	40.2	48.5	47.1	0.326
Discordance	36.3	70.9	56.9		58.8	60.2	45.1		59.8	51.5	52.9	

*p-value was obtained from the chi-square test for categorical variables

### Associations of nutrition knowledge, food availability, and food security with three patterns

The scores of “weight loss practices with weight dissatisfaction” pattern were not associated with nutrition knowledge, food availability, and food security. For only one question related to food availability (“I do not buy many fruits and vegetables because they cost too much.”), the percentage of individuals who agreed with this question significantly increased across the tertiles of pattern scores. The scores of “healthy eating behaviors” pattern were associated with high food availability but were not related to nutrition knowledge level and food security status. Individuals who had higher scores of “better knowledge of nutrition and health” pattern showed a significantly higher nutrition knowledge level compared to those who had lower scores (T3 vs. T1: 4.4 vs. 3.7 for mean number of corrected answer for nutrition knowledge questions). However, the scores of “better knowledge of nutrition and health” pattern did not show any associations with the questions for food availability and food security status (Table 4).

**Table 4:** Nutrition knowledge, food availability, and food security across the tertiles of scores of nutritional health attitudes and behaviors patterns

	Pattern 1				Pattern 2				Pattern 3			
	“Weight loss practices with weight dissatisfaction”				“Healthy eating behaviors”				“Better knowledge of nutrition and health”			
	Tertile1 (*n* = 102)	Tertile 2 (*n* = 103)	Tertile 3 (*n* = 102)	*p* -value*	Tertile1 (*n* = 102)	Tertile 2 (*n* = 103)	Tertile 3 (*n* = 102)	*p* -value*	Tertile1 (*n* = 102)	Tertile 2 (*n* = 103)	Tertile 3 (*n* = 102)	*p* -value*
Nutrition knowledge level (%)												
1–2	9.8	7.8	10.8	0.843	9.8	11.7	6.9	0.489	16.7	6.8	4.9	0.002
3–4	52.0	60.2	52.9		53.9	55.3	55.9		57.8	52.4	54.9	
5–6	34.3	26.2	31.4		33.3	27.2	31.4		22.6	36.9	32.4	
7–9	3.9	5.8	4.9		2.9	5.8	5.9		2.9	3.9	7.8	
Mean(SD) number of corrected answers	4.1 (1.4)	4.1 (1.4)	4.1 (1.4)	0.955	4.1 (1.4)	4.0 (1.4)	4.2 (1.3)	0.534	3.7 (1.3)	4.2 (1.3)	4.4 (1.4)	0.002
Food availability (%)												
I do not buy many fruits and vegetables because they cost too much.												
Agree	15.7	27.2	30.4	0.002	34.3	20.4	18.6	<0.001	27.5	25.2	20.6	0.457
Neutral	12.8	16.5	19.6		22.6	16.5	9.8		11.8	20.4	16.7	
Disagree	71.6	56.3	50.0		43.1	63.1	71.6		60.8	54.4	62.8	
I do not buy many fruits and vegetables because my family does not like them.												
Agree	4.9	2.9	2.0	0.620	5.9	3.9	0.0	0.013	2.9	3.9	2.9	0.620
Neutral	4.9	6.8	7.8		8.8	4.9	5.9		6.9	8.7	3.9	
Disagree	90.2	90.3	90.2		85.3	91.3	94.1		90.2	87.4	93.1	
The fresh produce in my area is usually high quality.												
Agree	60.8	60.2	71.6	0.229	53.9	55.3	83.3	<0.001	52.9	74.8	64.7	0.156
Neutral	31.4	31.1	20.6		34.3	35.0	13.7		37.3	19.4	26.5	
Disagree	7.8	8.7	7.8		11.8	9.7	2.9		9.8	5.8	8.8	
It is easy to buy food in my area.												
Agree	81.4	76.7	76.5	0.237	72.6	72.8	89.2	0.009	71.6	81.6	81.4	0.408
Neutral	13.7	16.5	13.7		18.6	18.5	6.9		22.6	11.7	9.8	
Disagree	4.9	6.8	9.8		8.8	8.7	3.9		5.9	6.8	8.8	
In minutes, how long does it take you to get to the grocery store?												
10 or less	48.0	50.5	47.1	0.831	47.1	42.7	55.9	0.855	53.9	48.5	43.1	0.626
20	26.5	23.3	23.5		27.5	27.2	18.6		19.6	23.3	30.4	
30	13.7	16.5	18.6		17.7	17.5	13.7		14.7	17.5	16.7	
45 or more	11.8	9.7	10.8		7.8	12.6	11.8		11.8	10.7	9.8	
How many times do you visit the grocery store in a month?												
0–1	3.9	2.9	2.9	0.531	2.0	3.9	3.9	0.616	2.9	4.9	2.0	0.766
2–4	52.9	46.6	50.0		47.1	55.3	47.1		48.0	51.5	50.0	
5 or more	43.1	50.5	47.1		51.0	40.8	49.0		49.0	43.7	48.0	
Food security status (%)												
High	62.8	46.6	48.0	0.088	50.0	48.5	58.8	0.101	48.0	45.6	63.7	0.077
Marginal	17.7	16.5	25.5		20.6	19.4	19.6		22.6	24.3	12.8	
Low	8.8	27.2	11.8		15.7	16.5	15.7		16.7	15.5	15.7	
Very low	10.8	9.7	14.7		13.7	15.5	5.9		12.8	14.6	7.8	

*p-value was obtained from the general linear model for continuous variables and the Mantel-Haenszel chi-square test for categorical variables

### Associations of overweight/obesity with three patterns

**Fig. 1:**
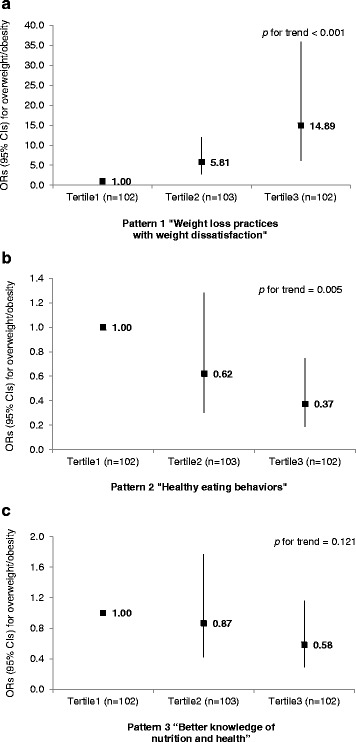
Multivariate odds ratios (ORs) and 95 % confidence intervals (CIs) for overweight/obesity across the tertiles of scores of nutritional health attitudes and behaviors patterns^1^. ^1^Multivariate logistic regression was performed to estimate the ORs, 95 % CIs, and *p* values for the prevalence of overweight/obesity across tertiles of pattern scores, taking the lowest tertile group as the reference group after adjustment for gender (male or female), age (<30y, 30–49y, or ≥ 50y), race/ethnicity (White/Caucasian, Black/African American, Hispanic/Latino, or other), marital status (single, married, cohabitating, or separated/divorced/widowed), and education level (≤ high school or ≥ associate’s degree/certificate or college)

The multivariate adjusted ORs and 95 % CIs for overweight/obesity across the tertiles of three pattern scores are presented in Fig. 1. After adjusting for potential confounding variables, the scores of “weight loss practices with weight dissatisfaction” pattern were positively associated with the prevalence of overweight/obesity (T2 vs. T1: OR = 5.81, 95 % CI = 2.81–12.05; T3 vs. T1: OR = 14.89, 95 % CI = 6.18–35.92, *p*-value < 0.001). The OR for overweight/obesity in individuals with the highest scores of healthy eating behaviors (pattern 2) was 0.37 (95 % CI = 0.19–0.75, *p*-value = 0.005) compared with those with the lowest scores as the reference group. However, one’s better knowledge of nutrition and health (pattern 3) was not associated with overweight/obesity.

## Discussion

Michigan MSHS child care providers in this study showed a high prevalence of overweight/obesity (overweight/obesity: 74 %, overweight: 24 % and obesity: 50 %, respectively), which was higher than the general population of US adults aged 20 years and over (overweight/obesity: 69 %, overweight: 34 % and obesity: 35 % in 2009–2012, respectively) [21] and lower than regular Head Start teachers in Texas (*n* = 181) (overweight/obesity: 79 %, overweight: 24 % and obesity: 55 % in 2008–2009, respectively) [8]. We found three patterns of nutritional health attitudes and behaviors among child care providers in Michigan MSHS centers. These patterns were influenced by sociodemographic characteristics, perception of weight status, nutrition knowledge, and food environments and associated with the prevalence of overweight/obesity.

This high risk of overweight/obesity among child care providers can be explained by their unhealthy attitudes and behaviors. Our data showed that individuals who tried to lose weight with dissatisfaction of weight had a high risk of overweight/obesity but individuals with high satisfaction of health and weight and healthy dietary behaviors showed a low risk of overweight/obesity. Teachers in Texas regular Head Start centers who were at a high prevalence of overweight/obesity had relatively low consumption of fruits and vegetables and high consumption of fried foods while they had dissatisfaction with their weight [8]. Teachers in German kindergarten reported several unhealthy behaviors, such as high screen time and low physical activity level and these behaviors were associated with a high risk of overweight/obesity [22]. However, specific information on nutritional health attitudes and behaviors and their association with weight status among MSHS child care providers is very limited despite their unique characteristics regarding sociodemographics, working hours and environments, children they serve, and knowledge about nutrition and health, which might be different from those of regular Head Start or kindergarten teachers.

In this study, nutritional health attitudes and behaviors of child care providers were influenced by their nutrition knowledge, perception of weight, food availability, and food security status. Nutrition knowledge among child care providers is limited and their personal dietary behaviors and food practices in classroom reflect this low level of nutrition knowledge [7, 8]. The insufficient nutrition knowledge and inaccurate perception of weight of child care providers may result in unfavorable health outcomes of themselves and children they serve. Lack of nutrition knowledge and cultural beliefs of Head Start staff were founded as important barriers to children’s healthy eating based on the 2008 cross-sectional study [23].

Our results, along with those of other studies, suggest that greater emphasis on developing and incorporating nutrition education or intervention targeting MSHS child care providers to ensure their health and wellbeing, as well as their ability to deliver MSHS programs to young children. Child care providers might have the important potential to influence nutritional health risks of children either directly or indirectly through transferring inappropriate attitudes and behaviors to children and providing misinformation and inappropriate advice and education [4]. Feeding behaviors of Head Start child care providers directly influenced on children’s food consumption [6]. However, there have been few studies that confirmed this important findings and interventions to promote nutrition knowledge and healthy dietary behaviors for child care providers at child care facilities [7, 8, 24]. Many efforts to reduce childhood overweight/obesity in Head Start programs have focused on family child care providers and their feeding methods [23, 25, 26]. According to a national survey of 1,583 Head Start centers in 2008, about 60 % held workshops to train new staff about feeding children and only 50 % offered workshops or activities for employees to improve their own eating behaviors [25].

In the current study, child care providers’ nutritional health attitudes and behaviors were determined by their socioeconomic status, perception of weight, nutrition knowledge, and food environments and needed to be changed to reduce the risk of overweight/obesity. According to our findings, the social cognitive theory-based intervention on dietary behaviors and nutrition knowledge can be suggested to expand knowledge on nutrition, health, and weight perception and improve eating behaviors of MSHS child care providers. This theory incorporates the interdependent relationships between personal, behavioral, and environmental factors to explain healthy eating behaviors [27]. The theory has widely been used for nutrition and/or health related interventions [28–31]. This approach might be an effective way to help MSHS child care providers to have healthy body image and appropriate weight control practices, support healthy dietary behaviors, and build nutrition knowledge and awareness of the importance of nutrition and health. Considering the fact that high proportion of Latino/Hispanic ethnicity among MSHS child care providers, culturally relevant and specific tailored interventions are also needed.

Findings of our study offer many opportunities and directions for future studies for this hard-to-reach and high risk group in the US although there are several limitations. The present study was based on a local-specific small-scaled and one year needs assessment investigation. Thus, our findings need to be confirmed through large-scaled studies at state-wide or national levels. This study relied on self-administered data from MSHS child care providers, which may influence the extent to which child care providers accurately report their nutritional health attitudes and behaviors and weight status although we trained directors of each center to guide their child care providers about the procedures of the survey. The survey questionnaire used in this study was not validated in MSHS child care providers, but each part of survey questionnaire was adopted from the validated questionnaires in low-income, minority populations at the fourth-grade reading level. To our knowledge, this study is the first attempt to examine nutritional health attitudes and behaviors and their associations with overweight/obesity among child care providers in MSHS centers, who are responsible for educating children and parents from MSFW families at a high risk for nutritional health problems.

## Conclusions

In conclusion, child care providers working in Michigan MSHS centers had specific nutritional health attitudes and behaviors associated with the prevalence of overweight/obesity. The current study supports that additional health interventions, including nutrition education targeting MSHS child care providers are urgently necessary. This might be helpful to improve their own health as well as to enhance their role as role models and educators in child care settings. Furthermore, these efforts for child care providers may be an efficient and effective non-invasive approach for reaching and helping large numbers of young children enrolled in MSHS programs. This practice has a great potential to decrease the growing gap in health disparity between the majority population and vulnerable group to nutritional health risk, such as MSFW families. Further studies are needed to focus on evaluating the effects of health interventions targeting MSHS child care providers on children’s health status.

## Abbreviations

BMI, body mass index; CI, confidence interval; MSFW, migrant and seasonal farm worker; MSHS, migrant and seasonal head start; OR, odds ratio; SD, standard deviation.
